# Toric phakic IOLs in keratoconus—evaluation of preoperative parameters on the outcome of phakic anterior chamber lens implantation in patients with keratoconus

**DOI:** 10.1007/s00417-020-05041-8

**Published:** 2021-01-19

**Authors:** Isaak R. Fischinger, Jascha Wendelstein, Kristin Tetz, Matthias Bolz, Manfred R. Tetz

**Affiliations:** 1 Department of Ophthalmology, Augentagesklinik Spreebogen Berlin, Alt-Moabit 101, 10557 Berlin, Germany; 2 Department for Ophthalmology and Optometry, Kepler University Hospital GmbH, Johannes Kepler University Linz, Linz, Austria

**Keywords:** Keratoconus, Phakic-IOL, Pellucidal marginal degeneration, Corneal crosslinking

## Abstract

**Purpose:**

To evaluate the influence of the type of the keratectasia and preoperative keratometry readings on the efficacy of implantation of iris-fixated phakic anterior chamber intraocular lenses (pIOL) in patients with keratoconus.

**Methods:**

In this retrospective study, iris-fixated pIOLs (Artisan/Artiflex (Ophtec®), Verisyse/Veriflex (AMO®)) were implanted in 38 eyes of 22 patients with stable keratoconus. Thirty-six eyes underwent corneal crosslinking (CXL) prior to the lens implantation. The refractive outcome was evaluated 6 weeks postoperatively and the influence of preoperative refraction and topo- and tomographical factors were analyzed.

**Results:**

The mean postoperative uncorrected distance visual acuity (UDVA_post_) was 0.25 ± 0.15 logMAR and was not statistically different from the mean preoperative corrected distance visual acuity (CDVA_pre_), which was 0.24 ± 0.13 logMAR. Twenty-seven eyes (71%) reached UDVA_post_/CDVA_pre_ ≥ 1 (efficacy index), whereas patients with PMD-like ectasia (*n* = 14) showed significantly (*p* = 0.003) higher efficacy index (100%) than patients diagnosed with keratoconus (*n* = 24) (54%). Higher eccentricity of the maximum posterior elevation showed a significant beneficial influence on the efficacy index (*p* = 0.021). Furthermore, a higher Amsler-Krumeich stage and preoperative MAE were correlated with a worse UDVA_post_. The mean absolute spherical equivalent was significantly decreased from 5.71 ± 4.96 D to 1.25 ± 1.20 D (*p* < 0.001). No significant difference was found in endothelial cell count.

**Conclusion:**

The results indicate that the implantation of phakic iris-fixated anterior chamber IOLs is a reasonable refractive option for patients with keratoconus. Keratoconus patients with a pellucidal marginal degeneration (PMD)-like appearance ectasia seem to benefit most from such procedures.

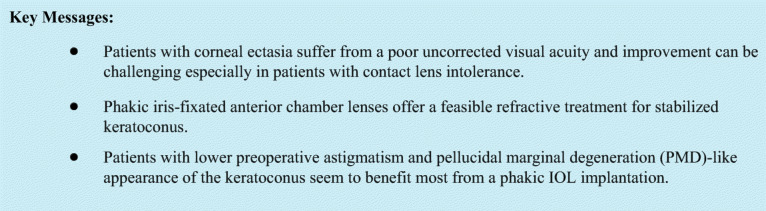

## Introduction

Keratoconus is a progressive disease with architectural alterations in the cornea, such as thinning and irregular bulging forward, which results of changes in the cornea’s biomechanical properties [1, 2]. From a refractive perspective, keratoconus can be characterized by astigmatism and higher-order aberrations such as coma and others. Pellucid marginal degeneration (PMD) is a much rarer disease, which is characterized by a more inferior peripheral thinning that results in a protrusion above the thinning and a crab-claw pattern on the topography map [3, 4]. The predominant optical error is astigmatism with less higher-order aberrations than keratoconus. Differentiating between keratoconus and PMD from the refractive properties can be difficult as the transition of aberrations seems smooth; therefore, terms as PMD-like ectasia or inferior keratoconus arose to describe those hybrid types [3, 5, 6]. Visual rehabilitation by contact lenses or spectacles is challenging in both types of keratectasia since they are associated with a variety of refractive errors due to the irregular topography [7]. Prior to the introduction of corneal crosslinking (CXL) by Seiler et al. in 1996, the only available treatment in the course of these progressive diseases was a penetrating keratoplasty with higher risks of loss of visual acuity or severe complications [8]. Since the progression of keratectasias can now be stopped in most cases, the demand of a reasonable refractive treatment for the increasing population of patients with stable keratectasia is substantial, in particular, if contact lens intolerance is present. Corneal refractive procedures are removing tissue and consecutively inducing a further weakening of the cornea and should therefore be avoided in these cases [9, 10]. Recently, an alternative approach is the use of phakic IOLs as a refractive treatment for stable keratectasia came up, however, these lenses were originally only designed for the treatment of regular astigmatism [11].

The previously published results of small sample size studies showed the potential of pIOLs to improve uncorrected visual acuity of patients with keratectasia. However, none of them assessed the influence of the type and level of keratectasia on the refractive success [12–15].

The aim of this retrospective study is to evaluate the influence of the type and stage of the keratectasia and keratometry readings on the refractive outcome after implantation of pIOLs.

## Patients and methods

This retrospective study includes a total of 38 eyes of 22 patients suffering from stable keratectasia, all receiving iris-fixated pIOLs between 2011 and 2018 in our clinic. The study protocol was approved by the ethical board of the institution and the local ethics committee (Berliner Ärztekammer, Eth-36/19) and was conducted according to the principles of the Declaration of Helsinki. The entire study data was collected from the data of the Augentagesklinik Spreebogen Berlin.

The inclusion criteria were stable keratoconus, absence of earlier refractive treatment, clear cornea, minimum anterior chamber depth, and age-dependent endothelial cell count as recommended by the manufacturer, age over 18, and well-documented data of visual acuity. All patients gave written informed consent after being provided a detailed description of the nature of the treatment.

Thirty-six of 38 eyes underwent corneal crosslinking using the classical Dresden protocol with 3 mW/cm^2^ in the past to halt progression of the corneal ectasia. The interval between the two procedures was 12 months in average.

**Fig. 1:**
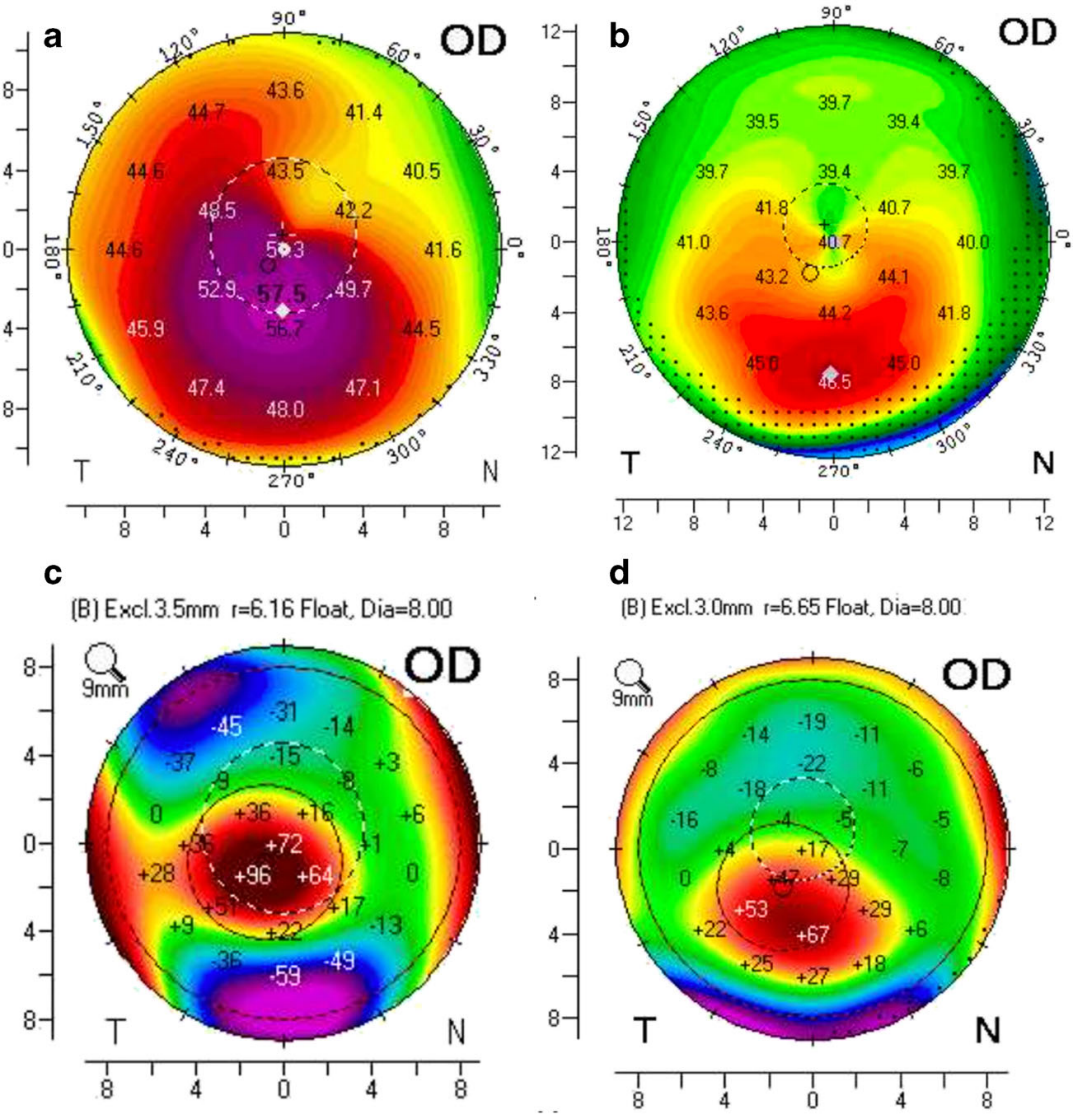
Scheimpflug imaging (Pentacam HR) of two different patients. **a** Sagittal curvature front of a classic keratoconus (patient A). **b** Sagittal curvature front of a PMD-like ectasia (patient B). **c** Posterior elevation map with a central maximal posterior elevation of the patient A. **d** Posterior elevation map with a slightly inferior maximal posterior elevation of the patient B. (PMD, pellucidal marginal degeneration)

The grading of the keratectasia and the differentiation between the subgroups was based on tomography imaging. The staging was done following the classical Amsler-Krumeich grading system [16, 17]. To differentiate between keratoconus and PMD-like ectasia, the eccentricity of the maximum posterior elevation and pachymetry as well as the topographical appearance were evaluated (Fig. 1). An eccentricity of the maximum posterior elevation of less than 1.5 mm suggested a keratoconus, 1.5–2.8 mm a PMD-like ectasia and > 2.8 mm a PMD [5].

### Preoperative and postoperative measurements

Preoperative measurements included manifest refraction, corrected distance visual acuity (CDVA), slit-lamp examination, dilated fundoscopy, pupillometry, tonometry, endothelial cell count, anterior chamber depth, and corneal tomography using HR Pentacam® (Oculus®, Wetzlar, Germany). Postoperative follow-up visits were conducted 1 day, 1 week, and 6 weeks postoperatively. Preoperative data was compared with the data collected 6 weeks postoperatively.

### Phakic intraocular lenses

Artisan (Ophtec®), Artiflex (Ophtec®), Verisyse (AMO®), and Veriflex (AMO®) lenses were implanted without brand preference depending on availability. Determination of the use of the rigid or flexible lens variants was based on the degree of myopia and astigmatism, as flexible lenses such as Artiflex and Veriflex are only available for a smaller range. The lens power was calculated by the manufacturer using the manifest refraction that rendered best visual acuity, *K* values, and anterior chamber depth.

### Surgical procedure

All treatments were performed by a single experienced surgeon (M.T.). The patient was prepared for the procedure with a peribulbar block and topical anesthesia. After pupillary constriction using pilocarpine and/or acetylcholine (Miochol®), a viscoelastic (Healon 5®) was introduced through a paracentesis. A 6.0 mm (Artisan/Verisyse 6), 5.0 (Artisan/Verisyse 5), or 3.2 mm (Artiflex) scleral incision was made at 12 o’clock to implant a rigid or flexible pIOL respectively. The lens was rotated to the desired position, and the haptics were fixated to the iris by enclavation of the iris fibers into the claw opening while the lens was held in place at the optic edge using special Artisan/Artiflex holding forceps. Thereafter, an iridotomy was performed with a pair of scissors, the viscoelastic was completely removed, and the self-sealing tunnel incisions were secured with 10–0 sutures. Postoperatively, antibiotic and steroid eyedrops (Isoptomax ®) were applied five times per day for 2 weeks, afterward tapered off over 2 weeks.

### Numerical evaluation

Descriptive analysis was performed for all variables in this study. Means and standard deviations of preoperative Scheimpflug imaging parameters (thinnest corneal thickness, *K*_max_, maximum posterior elevation (PE), eccentricity of PE, Qant, KI, and ISV) and demographic data as digital variables (age, eye) were documented or calculated. Multiple linear regression (backward elimination technique) was performed to detect any significant correlations between preoperative parameters and the outcome. All data were analyzed using Excel (2010, Microsoft Corp.) and SPSS (version 22.0, IBM Corp.). Statistical significance was assumed for *p* < 0.05.

## Results

Thirty-eight eyes of 22 patients (f:m = 10:12) were evaluated in this study. The average age was 33 years (range 20 to 47 years). Seventeen right and 21 left eyes were included.

The average Amsler-Krumeich staging was 1.76 ± 0.69 (range 1 to 3). Twenty-four eyes were diagnosed as keratoconus and 14 as PMD-like ectasia, none as classical PMD. Four patients did not show a distinct maximum posterior elevation in the inner 6.0 mm and therefore were not included in the evaluation of the posterior float. Table 1 shows the preoperative Scheimpflug parameters.

**Table 1:** Data from preoperative Scheimpflug imaging (Pentacam HR) of 38 eyes

Parameters	preoperative values (mean ± SD)
*K*_max_	51.92 ± 5.26
*K*1	43.9 ± 3.01
*K*2	47.0 ± 3.29
Astigmatism	3.23 ± 1.87
*R*_min_	6.61 ± 0.66
KI	1.20 ± 0.14
CKI	1.03 ± 0.03
IVA	22.31 ± 0.54
ISV	75.26 ± 39.15
IHD	0.0945 ± 0.0655
IHA	22.31 ± 17.77
Posterior float	
*n*	34 (90%)
Eccentricity of post float (mm)	1.23 ± 0.56
Amplitude (μm)	61.27 ± 28.4
Thinnest pachymetry (μm)	446.5 ± 45.4

*SD* standard deviation; *K*_*max*_ maximum anterior sagittal curvature; *Rmin* minimum radius of curvature; *KI* keratoconus index; *CKI* center keratoconus index; *IVA* index of surface asymmetry; *ISV* index of surface variance; *IHD* index of height decentration; *IHA* index of height asymmetry

**Fig. 2:**
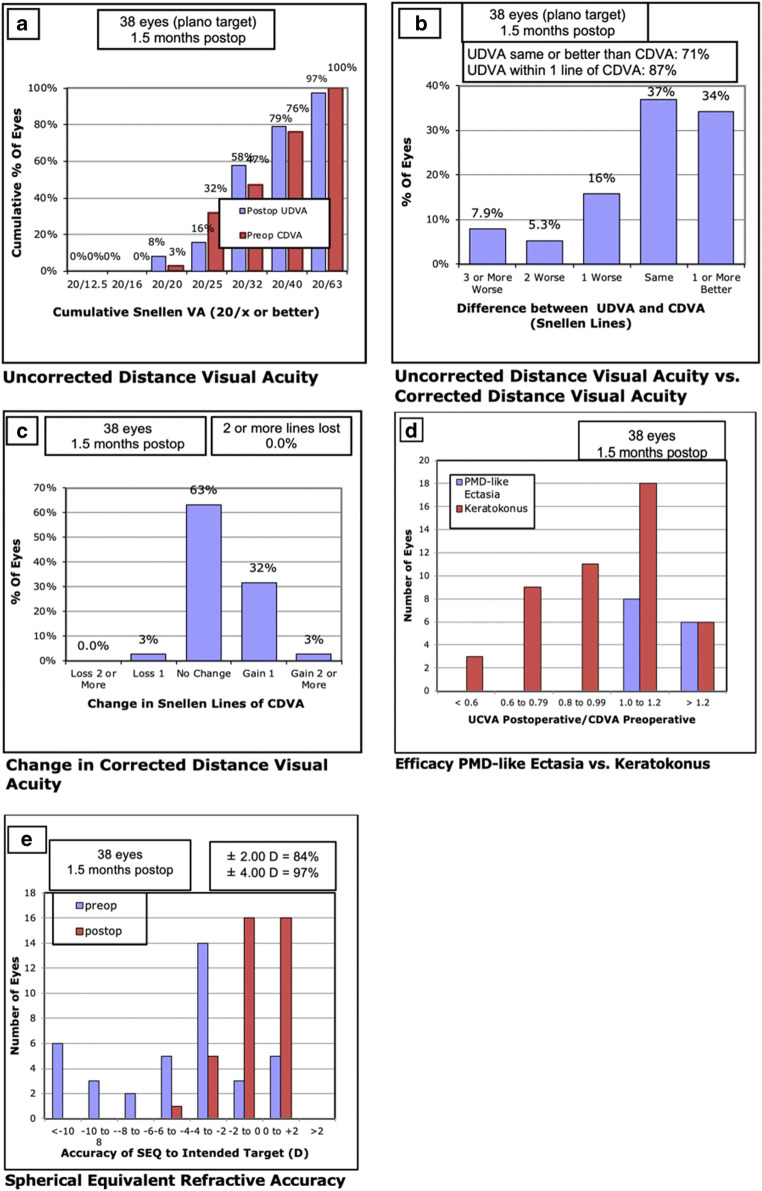
Standard graphs for reporting intraocular lens-based refractive surgery. **a** UDVA. **b** Difference between postoperative UDVA and preoperative CDVA. **c** Changes in CDVA. **d** Efficacy of patients with keratoconus versus PMD-like ectasia. **e** Accuracy of SE refraction (UDVA, uncorrected corrected distance visual acuity; CDVA, corrected distance visual acuity; PMD, pellucidal marginal degeneration; SE, spherical equivalent)

The postoperative uncorrected distance visual acuity (UDVA_post_) is shown in Fig. 2a. The mean UDVA_post_ was 0.25 ± 0.15 logMAR (mean decimal 0.58) and was not statistically different from mean corrected distance visual acuity (CDVA_pre_) which was 0.24 ± 0.13 logMAR (mean decimal 0.59) (*p* = 0.688). Seventy-nine percent of all patients reached a UDVA of 0.3 logMAR (decimal 0.5) and 13% of 0.1 logMAR (decimal 0.8) respectively.

The correlation of preoperative parameters with the UDVA_post_, the efficacy index (UDVA_post_/CDVA_pre_), and the number of eyes reaching an efficacy index ≥ 1 (UDVA_post_/CDVA_pre_ ≥ 1) is shown in Table 2. A higher Amsler-Krumeich stage and preoperative MAE were correlated with a worse UDVA_post_. No other preoperative parameters showed a significant influence on the UDVA_post_.

**Table 2:** Correlation between preoperative parameters and outcome

Parameters	UDVA_post_	Efficacy index (UDVA_post_/CDVA_pre_)	Efficacy index ≥ 1(UDVA_post_/CDVA_pre_ ≥ 1)
Diagnosis	*R* = 0.247	*R* = 0.394	*p* = 0.003
	*p* = 0.135	*p* = 0.046	
Amsler-Krumeich stage	*R* = −0.285	*R* = 0.093	*p* > 0.05
	*p* = 0.014	*p* = 0.580	
*K*_max_	*R* = −0.229	*R* = 0.037	*p* > 0.05
	*p* = 0.167	*p* = 0.826	
Astigmatism (Scheimpflug)	*R* = −0.195	*R* = 0.292	*p* > 0.05
	*p* = 0.240	*p* = 0.075	
*R*_min_	*R* = 0.196	*R* = −0.122	*p* > 0.05
	*p* = 0.227	*p* = 0.465	
KI	*R* = −0.248	*R* = 0.218	*p* = 0.083
	*p* = 0.134	*p* = 0.189	
CKI	*R* = −0.161	*R* = 0.040	*p* > 0.05
	*p* = 0.334	*p* = 0.810	
IVA	*R* = −0.242	*R* = 0.250	*p* = 0.072
	*p* = 0.143	*p* = 0.130	
ISV	*R* = −0.292	*R* = 0.219	*p* > 0.05
	*p* = 0.075	*p* = 0.187	
IHD	*R* = −0.324	*R* = 0.162	*p* > 0.05
	*p* > 0.05	*p* = 0.330	
IHA	*R* = −0.171	*R* = 0.104	*p* > 0.05
	*p* = 0.305	*p* = 0.534	
Posterior float			
Eccentricity of posterior float	*R* = 0.308	*R* = 0.318	*p* = 0.021
	*p* = 0.076	*p* = 0.067	
Amplitude	*R* = −0.107	*R* = 0.255	*p* > 0.05
	*p* = 0.546	*p* = 0.145	
Thinnest pachymetry	*R* = 0.338	*R* = −0.078	*p* > 0.05
	*p* > 0.05	*p* = 0.639	
MAE	*R* = −0.303	*R* = −0.187	*p* > 0.05
	*p* = 0.006	*p* = 0.261	
Sphere (subjective refraction)	*R* = 0.282	*R* = 0.284	*p* > 0.05
	*p* = 0.86	*p* = 0.084	
Astigmatism (subjective refraction)	*R* = 0.033	*R* = −4.59	*p* > 0.05
	*p* = 0.844	*p* = 0.019	

*UDVA*_*post*_ postoperative uncorrected distance visual acuity; *CDVA*_*pre*_ preoperative corrected distance visual acuity; *R* correlation coefficient; *K*_*max*_ maximum anterior sagittal curvature; *R*_*min*_ minimum radius of curvature; *KI* keratoconus index; *CKI* center keratoconus index; *IVA* index of surface asymmetry; *ISV* index of surface variance; *IHD* index of height decentration; *IHA* index of height asymmetry; *MAE* mean absolute spherical equivalent

The diagnosis PMD-like ectasia (*p* = 0.046) and a lower preoperative astigmatism (*p* = 0.019) in the manifest refraction were the only parameters significantly correlated with a higher efficacy index. A higher eccentricity of the maximum posterior elevation was a significant predictive factor for a higher number of eyes achieving an efficacy index ≥ 1 (*p* = 0.021). None of the other parameters showed a significant influence on the efficacy index.

Efficacy is shown in Fig. 2b. Fourteen patients (34%) showed a better UDVA_post_ than CDVA_pre_. Twenty-seven eyes (71%) reached a UDVA_post_/CDVA_pre_ ≥ 1, all eyes (100%) with the diagnosis PMD-like ectasia but only 13 eyes (54%) with keratoconus reached a UDVA_post_/CDVA_pre_ ≥ 1, which illustrates a significant difference between the two groups (*p* = 0.003). The efficacy index of eyes with keratoconus versus PMD-like ectasias is shown in Fig. 2d.

The mean absolute spherical equivalent (MAE) was significantly reduced from 5.71 ± 4.96 D to 1.25 ± 1.20 D (*p* < 0.001). The spherical equivalent refractive accuracy is shown in Fig. 2e. Safety is shown in Fig. 2c. Only one eye lost one line. Endothelial cell count (ECC) was 2728 ± 235 cells/mm^2^ pre- and 2778 ± 142 cells/mm^2^ postoperatively (*p* = 0.548). ECC could only be measured in 24 eyes postoperatively due to loss of follow-up.

## Discussion

The major findings of this study are the following:

- Phakic iris-fixated anterior chamber lenses offer a feasible refractive treatment for stabilized keratoconus.
- The UDVA after pIOL implantation is equal or better than the preoperative CDVA in 71% and better in 34% in eyes with keratoconus. None of the patients lost two lines.
- Advanced keratoconus and a high MAE are correlated with a worse postoperative UDVA.
- The implantation of pIOLs shows better refractive results in patients with PMD-like ectasia than in patients with classic keratoconus.

Ever since the progression of keratoconus can be stopped by CXL [8], there is an increasing, relatively young population with the desire to improve UDVA by refractive treatment. Glasses only allow limited visual acuity in these cases; and if the patient develops contact lens intolerance, there have been little options for visual rehabilitation until now. Topography-guided laser ablation combined with CXL had been performed in several cases [18–20]. However, corneal laser ablation increases the risk of progressive corneal ectasia in eyes with keratoconus, especially when higher corrections are needed and is therefore considered contraindicated or at least highly controversial [9, 10]. Customized crosslinking shows promising results in terms of regularization of the cornea, but the technology cannot correct higher refractive errors [21].

Although the implantation of pIOLs was not recommended for keratectasias in the first place and the lens manufacturers proclaimed in their exclusion criteria that the lenses were meant for regular astigmatism only [11], favorable results in a few case studies have been achieved by implantation of pIOLs and ICLs in stable keratectasia in recent years [12. 13, 22]. Izquierdo et al. [12] reported the implantation of anterior chamber phakic Artiflex lenses in 11 eyes with grade I or II keratoconus 6 months after CXL. Only spherical Artiflex lenses were used, but postoperative UDVA of 0.3 logMAR or better was observed in all patients. More recently, Güell and coworkers published a study including 17 eyes with progressive grade I or II keratoconus with regular central astigmatism receiving pIOL implantation 3 months after CXL [13]. The efficacy in terms of postoperative UDVA versus preoperative CDVA was comparable with our results. A total of 94.1% reached a postoperative UDVA of 0.3 logMAR or better, which was also the minimum reached CDVA preoperatively. Hashmani S et al. reported a case of bilateral Artiflex implantation 4 months after CXL achieving bilateral postoperative UDVA of 0.3 logMAR equivalent to preoperative CDVA [14]. Fadlallah A et al. reported significantly improved UDVA and SE after ICL implantation in 16 eyes 6 months after CXL [22].

To our knowledge, none of these or any other studies so far have evaluated the influence of preoperative factors on the refractive outcome of the procedure. But hardly surprising, especially the phenotype of the keratectasia seems to have a relevant influence on the efficacy. Implantation of pIOLs in patients with PMD-like ectasia showed significantly better efficacy (UDVA_post_/CDVA_pre_) than in patients with classical keratoconus. This might be due to the higher degree of regular central astigmatism in patients with PMD or PMD-like ectasia, as pIOLs only allow for correcting regular astigmatism. Accordingly, spectacle correction works better in PMD than in keratoconus [23]. Therefore, the implantation of pIOLs can particularly be recommended in PMD-like ectasia and most probably PMD. Still, our results for keratoconus patients are pleasant and a success for patients as well. Our knowledge from this study is most likely valid for ICL implantation in keratectasia too and might support the preoperative planning of cataract surgery and ease the selection of (pseudophakic) toric IOLs.

The Amsler-Krumeich stage of the keratectasia did not have an impact on the efficacy of the intervention, but the postoperative UDVA was significantly worse in progressed keratectasia (higher Amsler-Krumeich stage) going along with high MAE. Worse results for patients with higher preoperative refractive error were found for pIOL implantation for myopia earlier [23].

Dick et al. reported UDVA after pIOL implantation for myopia in 290 eyes of 0.3 logMAR or better in 97.2% [24]. A multicenter study with 662 eyes with moderate to high myopia enrolled found a postoperative UDVA of 0.3 logMAR in 84% which is only slightly better than our results (79%) [25]. This comparison indicates that a reasonable UDVA can be reached quite reliably for patients suffering from mild to moderate corneal ectasia too.

The timing of the implantation of the pIOL should be discussed critically. Some authors suggested to wait 3 or 6 months after CXL before implanting the pIOL [12, 13]. In our study, the average interval between the two procedures was 12 months. We believe a minimum interval of 6 months should be maintained to have a more stable preoperative refraction, as most flattening is observed rather early after CXL [26]. However, few cases with continuous flattening after CXL could suffer from later occurring refractive changes [27]. Such cases could not be detected in this study, as we monitored the early postoperative phase only.

The validity of this study might be affected by the high standard deviation of the Scheimpflug imaging in progressed keratectasia and the sample size which still to our knowledge is the biggest group published. Furthermore, long-term follow-up and longer intervals between CXL and pIOL implantation might be favorable in terms of ongoing flattening due to CXL. Additional surgical effort for future surgeries such as cataract surgery or keratoplasty where the pIOL has to be removed should be discussed with the patient and kept in mind by the surgeon.

In conclusion, this study shows that the implantation of pIOLs is a valuable procedure for visual rehabilitation in eyes with keratectasia. Patients with more eccentric ectasia and decentered maximum posterior elevation as it is present in PDM-like ectasia seem to benefit the most from such a procedure.
